# The Endoscopic Resection of Sellar and Suprasellar Epidermoid Cyst in a Pediatric Patient: A Case Report and Review of the Literature

**DOI:** 10.7759/cureus.50084

**Published:** 2023-12-06

**Authors:** Othman Alhammad, Faisal Joueidi, Hanan N Aljohani, Abdulaziz A Basurrah, Muhammad Ansary

**Affiliations:** 1 Neurosurgery, King Faisal Specialist Hospital and Research Centre, Riyadh, SAU; 2 College of Medicine, Alfaisal University, Riyadh, SAU; 3 Neurosurgery, King Abdullah Medical City in Holy Capital, Makkah, SAU; 4 Neuroscience Centre, King Faisal Specialist Hospital and Research Centre, Riyadh, SAU

**Keywords:** case report, skull base, pediatrics, brain neoplasm, suprasellar region, sellar region, epidermoid cyst

## Abstract

Epidermoid cysts are benign congenital tumors that originate from the ectodermal tissue. The sellar/suprasellar region is an infrequent location for epidermoid cysts and such cases are rarely reported in pediatric patients, as these become symptomatic only when they reach 30 years of age. Surgical intervention is considered the ideal treatment option in patients with suprasellar epidermoid cysts, either via open or endonasal approach. We discuss a case of a 12-year-old male who presented with left visual impairment and was treated with successful resection through an endoscopic endonasal approach (EEA). We also engage in a literature review of the use of EEA in the management of sellar/suprasellar epidermoid cysts in the pediatric age group.

## Introduction

Epidermoid cysts are benign slow-growing tumors that arise from the remnants of ectodermal cells [1]. Epidermoid cysts are rare lesions that are mainly located in the intracranial space, accounting for 0.5-1.8% of all intracranial tumors. However, it is rarely reported in pediatric patients [1,2]. Between 40 and 50% of intracranial epidermoid cysts affect the cerebellopontine cisterns, and 10-15% affect suprasellar spaces or the middle cranial fossa [3]. Because of the slow-growing nature of these tumors, sellar epidermoid cysts are usually asymptomatic in pediatric patients [4]. Surgical intervention is considered the ideal treatment method for sellar/suprasellar cysts, and multiple options are available including the endoscopic endonasal approach (EEA) or open technique [4]. However, the procedure can be challenging due to the involvement of vital neurovascular structures [1].

## Case presentation

**Figure 1:**
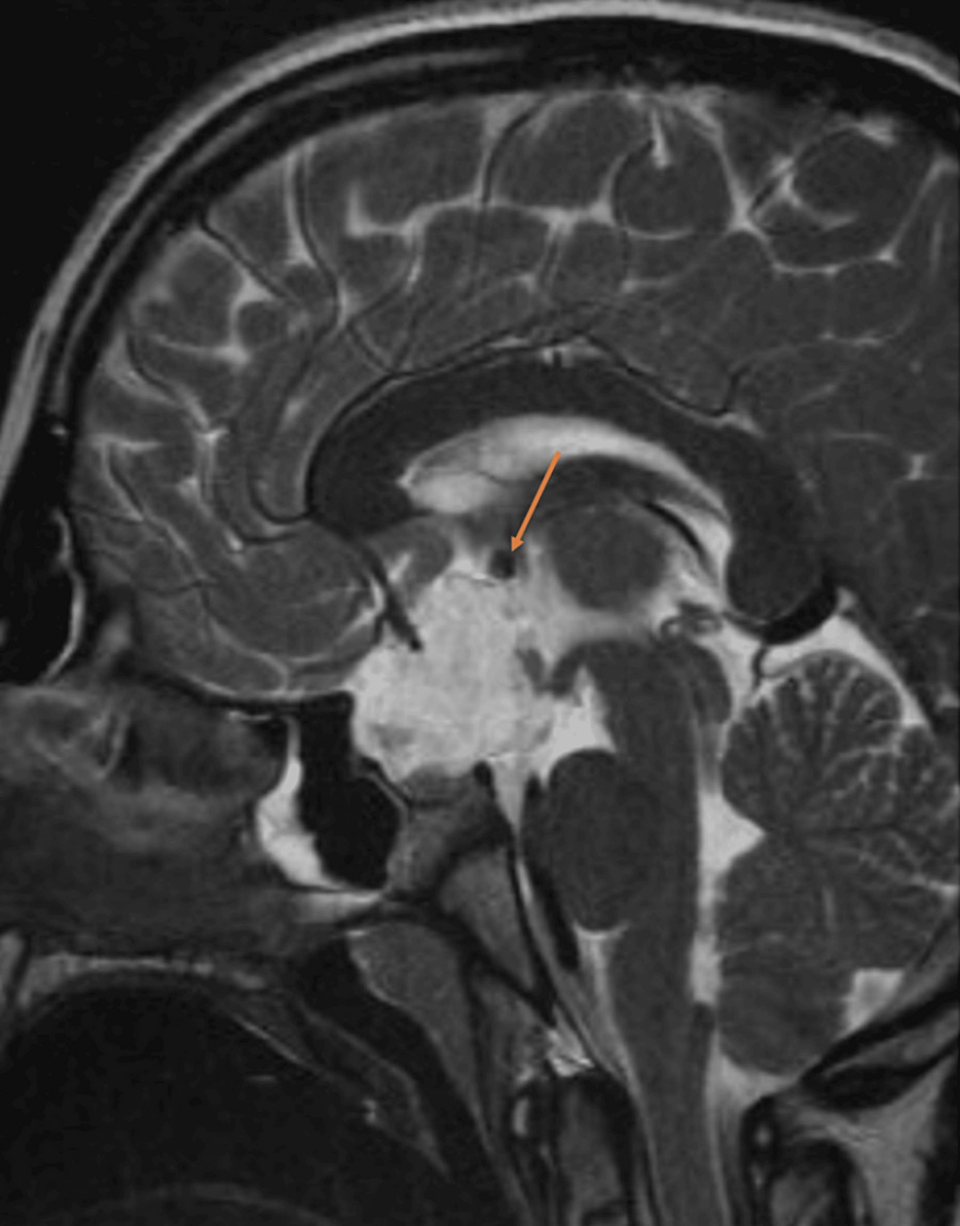
Preoperative sagittal T2 image Suprasellar mass measuring 30 x 23 x 27 mm; there is a residual mass effect on the adjunct structures manifested by the compression of the pituitary gland which is effected against the sellar floor remodeling of sellar tuberculum, expansion, and stretching of the circle of Willis

**Figure 2:**
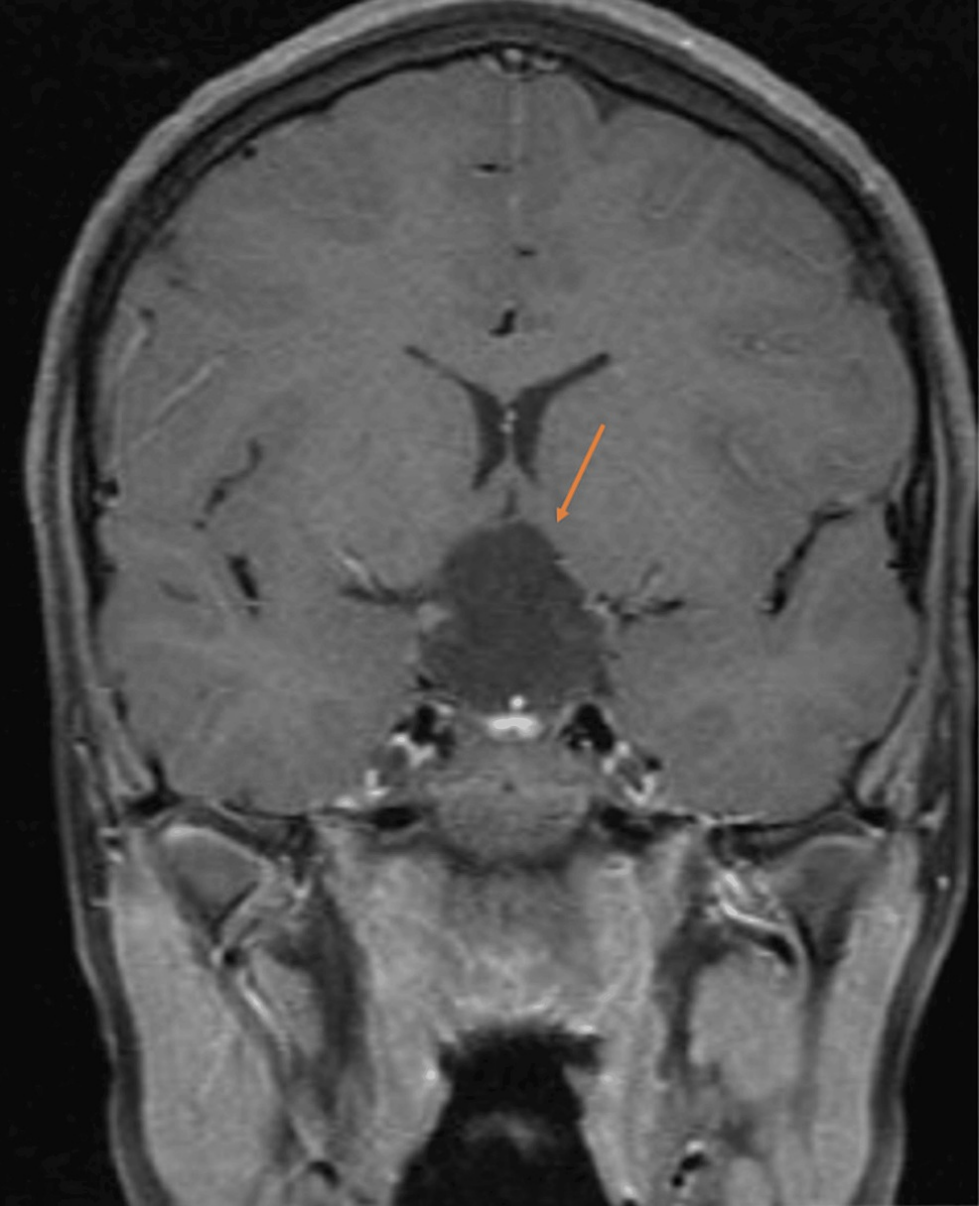
Preoperative coronal T1 image Suprasellar mass measuring 30 x 23 x 27 mm; there is a residual mass effect on the adjunct structures manifested by the compression of the pituitary gland which is effected against the sellar floor remodeling of sellar tuberculum, expansion, and stretching of the circle of Willis. Also, there is lateral displacement of bilateral optic tracts and chiasm superior and posteriorly

**Figure 3:**
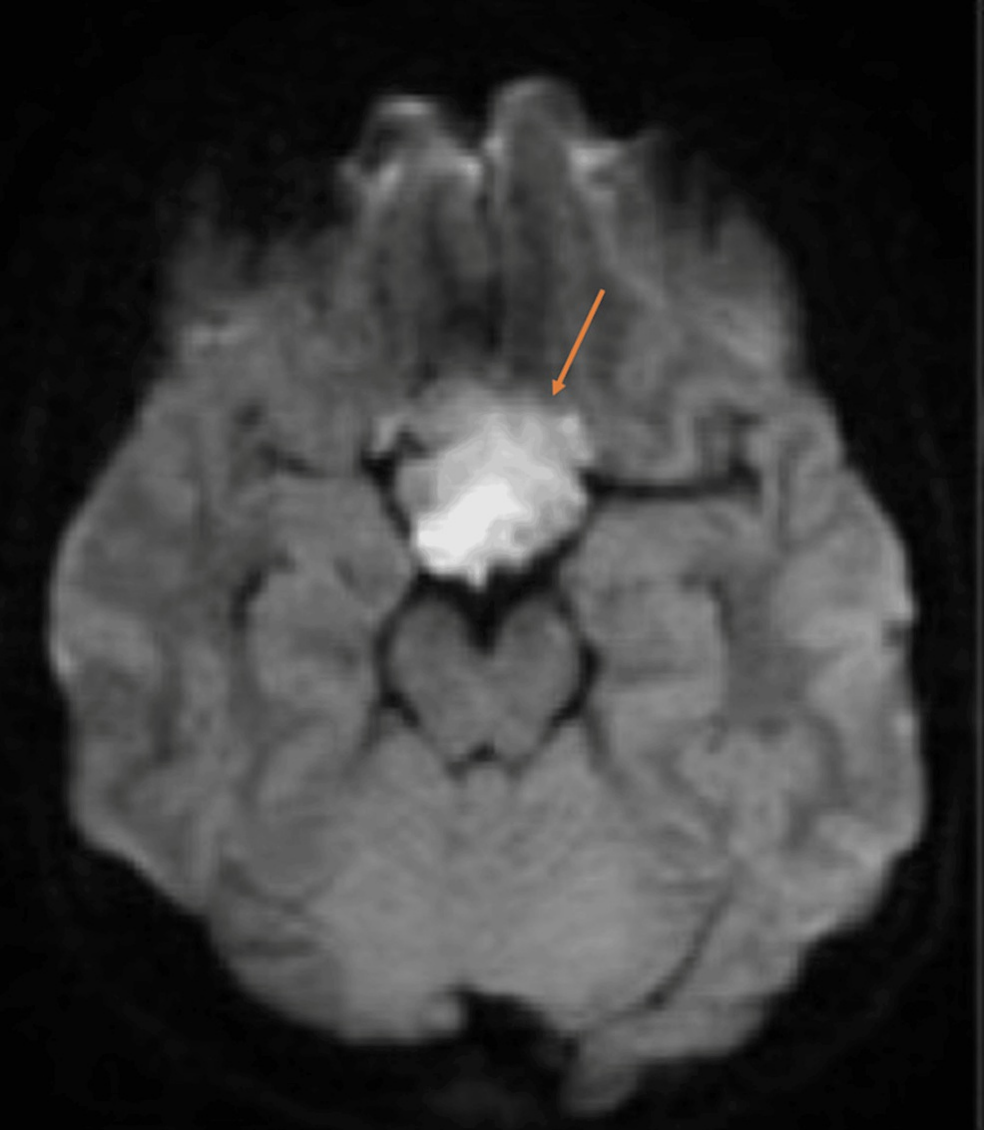
Preoperative axial diffusion-weighted image (DWI) Mild diffusion restriction with a heterogeneous nulling signal on FLAIR

The patient was a 12-year-old male who was medically healthy and presented with slowly progressive visual impairment for one year. Ophthalmology examination showed bitemporal hemianopia denser on the left eye and decreased visual acuity in the right eye (20/22) and left eye (20/300). A dilated fundus exam showed a bilateral pale optic disk in the temporal OS. The patient had normal extraocular movement and no nystagmus or diplopia; other cranial nerve examinations were unremarkable and motor and sensory examinations were normal. Laboratory investigation and hormonal profile were unremarkable (FSH: 2.9, LH: 3.0, FT4, T3: 2.3, TSH: 2.14, ACTH: 22.1, cortisol: 231, and prolactin: 14.60). MRI of the brain showed suprasellar heterogeneous mass measuring 30 x 23 x 27 mm with mass effect manifested by the compression of the pituitary gland against the sellar floor with mild diffuse restriction (Figures 1-3).

**Figure 4:**
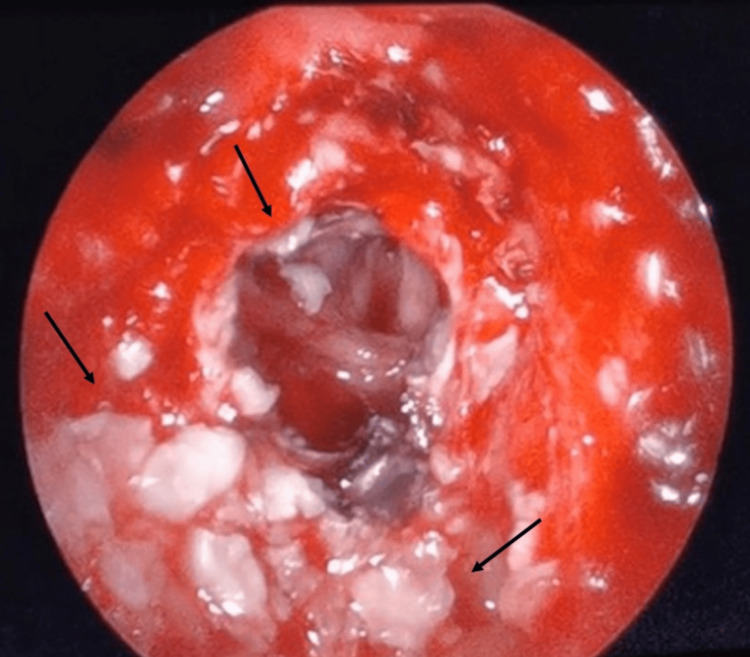
Intraoperative image The image shows the classical appearance of an epidermoid cyst. There is a glistening pearly white surface measuring 4 x 3 x 0.6 cm, rich in cholesterol crystals with focal calcifications

**Figure 5:**
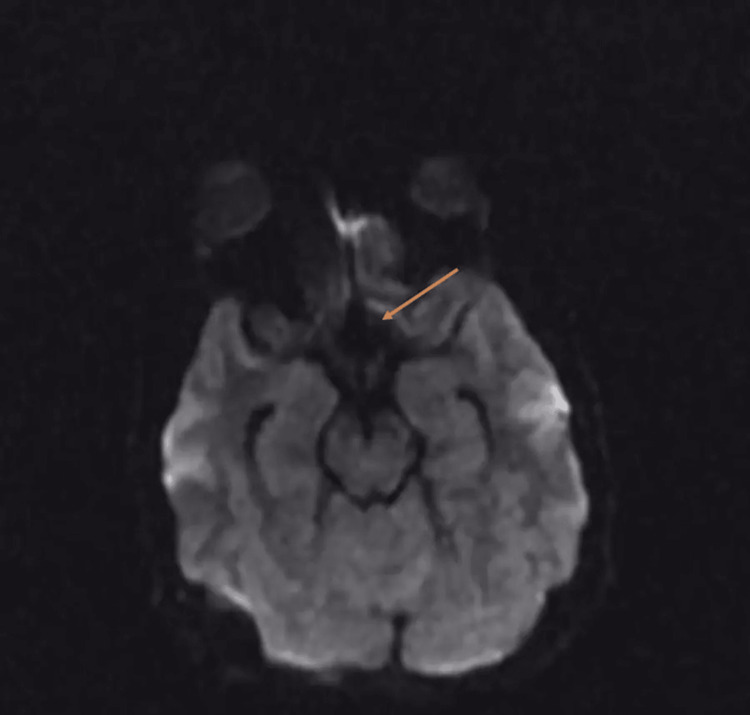
Postoperative axial diffusion-weighted image There is no diffusion restriction in the sellar/suprasellar area

**Figure 6:**
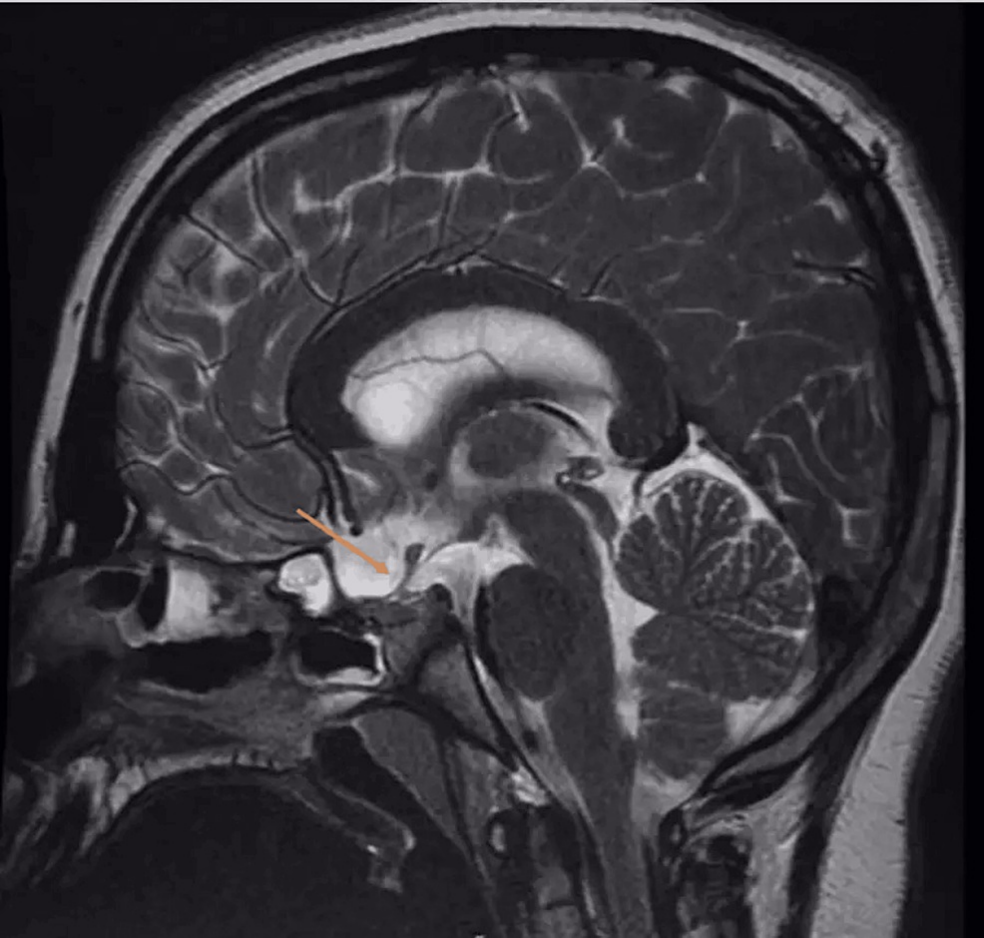
Postoperative coronal T1 image The image shows ectasia of the sellar/suprasellar area due to chronic mass effects, and intact pituitary stalk and gland

Accordingly, the patient was scheduled for surgery. An endoscopic endonasal transsphenoidal approach with partial middle turbinectomy for sellar epidermoid cyst excision was employed. The optic nerve and chiasm were visualized; the left optic nerve was affected more, and the resection of the cyst and decompression of the optic nerve and chiasm were achieved. The gross total resection (GTR) was achieved uneventfully (Figure 4). The repair was then performed in two layers, first using Tutoplast 4 x 4 cm, and the left nasoseptal flap was elevated and placed over the repair. Postoperatively, the patient developed central diabetes insipidus, and the vision in the left eye (the same eye that was affected preoperatively) deteriorated, from 20/300 to light perception only. Hence, the patient was given desmopressin; steroids were not used, and the patient was scheduled to regularly follow up after one year with the endocrinology team. One year later, the patient's left vision did not improve. However, his hormonal profiles (FT4: 13.7, TSH: 1.55, ACTH: 19, cortisol: 436, prolactin: 0.42) were within the normal range. There was no documented residual or recurrence of the epidermoid cyst. The postoperative axial diffuse-weighted image showed no diffusion restriction in the sellar/suprasellar area (Figure 5). Coronal T1 image showed ectasia of the sellar/suprasellar area due to chronic mass effects, and intact pituitary stalk and gland (Figure 6). Coronal T1 +C image showed no residual lesion and normal integrity of the optic chiasm.

**Figure 7:**
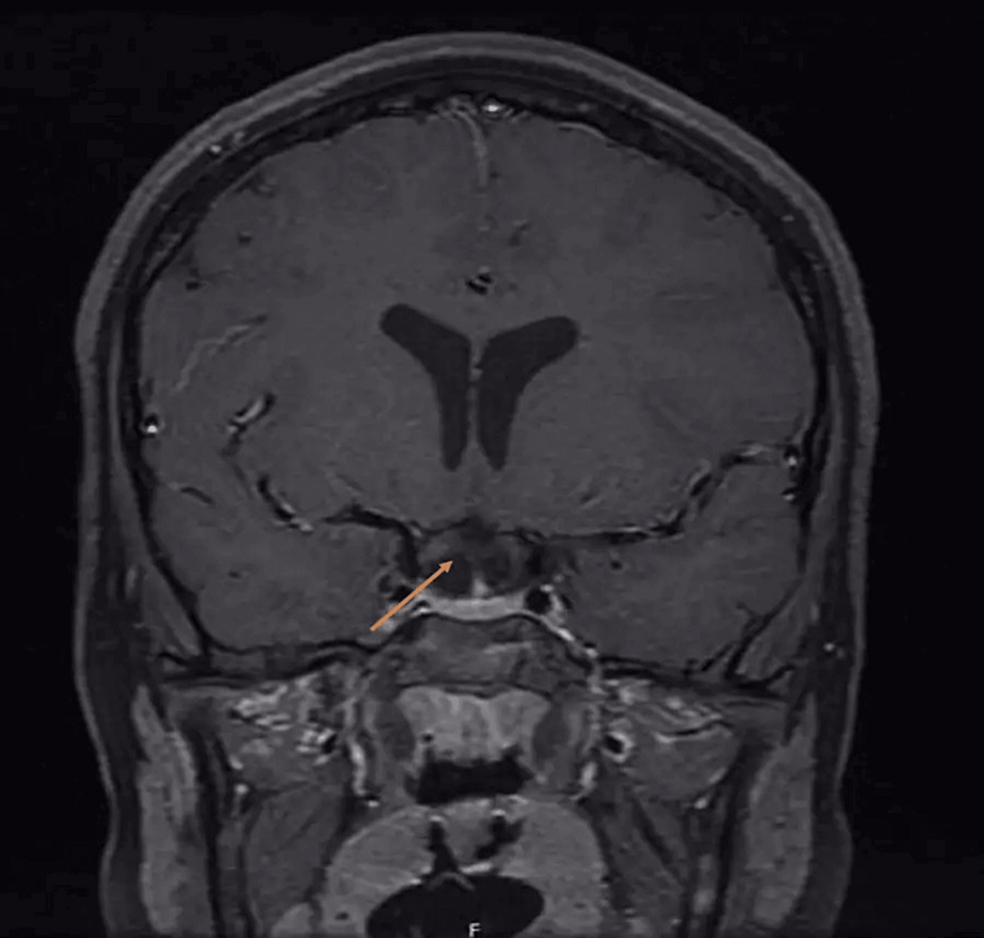
Postoperative coronal T1 +C image The image shows no residual lesion and normal integrity of the optic chiasm

## Discussion

An epidermoid cyst is a rare congenital benign cystic lesion that occurs due to the proliferation of multi-potent embryonic stem cells or lateral displacement of ectodermal cells [5]. The incomplete separation of the epidermal and neural ectoderm occurs between the third and fifth gestational week, resulting in the inclusion of epiblast in the neural tube that closes during the gestational period [4,6]. An epidermoid cyst is filled with keratin laminae, water, and lipids structures in its wall and covered by mature keratinized squamous epithelium [4,7]. The accumulation of keratin and cholesterol, which are breakdown products, can increase their tendency to grow [7]. Suprasellar epidermoid cysts and tumors usually vary in presentation depending on the age of the patient, location, extension, and progression into the surrounding structures [7]. In most cases, the presentation of epidermoid cysts is clinically silent [7]. However, symptomatic presentation can occur depending on the location of the tumor, cranial nerve involvement, or mass effect [5,7]. They have a slow rate of growth, due to which they usually become symptomatic only in the third or fourth decades of life. These tumors have an affinity for the basal cisterns, the suprasellar and cerebellopontine angle (CP) cisterns being the most common sites [8]. The symptoms most commonly pertain to the optic apparatus, such as visual impairment, optic atrophy, and bitemporal hemianopia [9]. Pituitary dysfunction may or may not be evident along with the visual symptoms [10].

Various imaging modalities are used to accurately diagnose sellar/suprasellar cysts. CT is considered the first-line modality; on CT, epidermoid cysts appear as hypodense, homogeneous, well-circumscribed, non-enhanced lesions, and extra-axial lesions [5]. However, in some cases, they may have a hyperdense appearance with calcified lesions (10% of cases). The hypodensity of the cyst is attributed to it being filled with keratin debris, water, and lipids [11]. Furthermore, the reduction of intensity enhancement is attributed to the low vascularity of the cyst [9].

MRI is the gold standard method employed to differentiate between epidermoid cysts from other intracranial tumors [5]. On MRI, the epidermoid cyst appears on T1 as a hypointense. Diffusion-weighted sequence usually has a hyperintense appearance with a restricted pattern [7]. Furthermore, it is considered both sensitive and specific in the diagnosis of epidermoid cysts when compared to other cystic tumors, due to the presence of debris, and proteins which can change the radiological findings from a homogeneous signal to a non-homogeneous signal [5]. The rare dense or white epidermoid appearance shows a reverse signal intensity on T1 and T2 images [5]. MRI can assess the depth extension and mass effect of the tumor on the neurovascular structures without interfering with the density of the skull base. Grossly, it is a well-delineated lesion that appears pale-uninoculated, and translucent with an irregular nodular shiny surface; focal calcification may also be present [4,12]. The cyst is filled with soft waxy material that is rich in cholesterol crystals. Histopathologically, they appear as pearly lesions lined with stratified squamous keratinizing epithelium and filled with keratin [7].

The treatment mainly involves surgical resection using different surgical approaches that may be tailored to the direction of growth of different suprasellar region tumors; for example, sub-frontal, front-lateral, interhemispheric open approaches, or transnasal endoscopic approach [2]. Total gross resection with the cyst wall is necessary to prevent recurrence [7]. However, the degree of resection is limited by the adherence of the surrounding structure [7]. This is due to the involvement of an inflammatory reaction that can occur when the content of the epidermoid cyst travels from the capsule to the subarachnoid space that contains vascular structures resulting in local adhesions [7]. The endonasal endoscopic transsphenoidal approach is more commonly used than craniotomy [4]. The advantage of this approach includes better cosmetic results, avoidance of neurovascular retraction, and less surgical length [4]. The endonasal endoscopic transsphenoidal approach is used to resect epidermoid cysts that are less than 4 cm in diameter and mainly located in the ventral aspect of the skull base [4]. Total resection of the cyst requires adequate exposure of the cyst to enable better capsular resection [4]. Capsular resection reduces the regrowth and malignant transformation of the cyst [4]. Subtotal or near-total resection is not recommended as it can result in the production of keratin that can progressively increase the size of the cyst due to the inflammatory response, which can lead to higher chances of recurrence and cystic rapture (between 27 and 33.4% of cases) [4]. Some surgeons may perform simultaneous cranionasal combined surgery [2].

Possible limitations of the endonasal endoscopic transsphenoidal approach in children include the narrow sino-nasal pathways and, until recently, the lack of dedicated surgical instruments [13]. Concerning the first issue, the binostril approach together with the use of 2.7-mm diameter optics and 3-mm diameter optics recently developed allows to overcome the problem of the small nostrils in several cases. However, the involvement of both nostrils does not increase the risk of nasal morbidity. As to the pneumatization of the sphenoid sinus, we did not observe an augmented risk of not/partially pneumatized sinus (only one conchal variant). Although traditionally considered to start from the third to the fourth year of age and to be completed during the second decade of life [14,15], several studies based on anatomical, CT scans, and MRI have demonstrated that the sphenoid pneumatization begins as early as the first months after birth and usually finishes within the first decade [13,16]. Since a long-term prognosis is expected, it is best to remove it to the maximum extent possible, if not the entire lesion, especially in children. In the case of recurrence, adhesions may occur, and extraction may be more difficult. In recent years, it has therefore been considered desirable to extract the lesion to the farthest extent [17,18,19].

Postoperative complications include endocrine disturbances; diabetes insipidus (DI) is a common complication that occurs in the parasaller region, indicating hydrocephalus and chemical meningitis that happens due to the leak of keratin debris into the cerebrospinal fluid (CSF) [4,7]. The prognosis is considered excellent in patients with epidermoid cysts, with a good long-term survival rate due to the recognition, growth pattern, and proper diagnosis of the condition [5]. A study by Cappabianca et al. showed that the endonasal endoscopic transsphenoidal approach is associated with reduced hospital length of stay, less operative time, and better outcomes in terms of postoperative mortality and morbidity. However, tumor recurrence is considered high in comparison with craniotomy [13]. Ikeuchia et al. have reported the case of a six-year-old female with progressive vision deterioration and bitemporal hemianopia. The patient underwent frontoparietal craniotomy and interhemispheric approach; postoperatively, the patient experienced worsening of the vision [20]. Eliash et al. have reported a case of a five-year-old child with an unusual suprasellar epidermoid cyst with pituitary insufficiency and optic nerve involvement. The patient underwent a frontal craniotomy [21].

Ahmad et al. reported a sellar case that had visual disturbance and developed hydrocephalus after aseptic meningitis; postoperatively, the patient underwent shunt placement [22]. Bhat et al. reported a case of a 17-year-old patient who presented with generalized tonic-clonic seizures and underwent frontal craniotomy and developed tumor recurrence postoperatively [23]. Symss et al. reported a case of a patient who experienced blurry vision, papilledema, unsteady gait, and hydrocephalus; the patient underwent frontoparietal craniotomy, interhemispheric approach, and, postoperatively, the patient developed chemical meningitis [8]. Another study by AlAbdullah et al. reported a patient with bilateral visual acuity deterioration [4]. The patient underwent endoscopic surgery with a transnasal transsphenoidal approach. Postoperatively, the patient experienced a partial recovery in visual acuity of the left eye. However, no improvement was noticed in the right eye [4]. In our case, the patient had left-sided visual impairment, and he underwent surgical intervention with an endonasal endoscopic transsphenoidal approach and GTR. Postoperatively, the patient's left-sided visual impairment deteriorated to light perception and central diabetes insipidus, which was managed medically. 

Table 1 presents a summary of all the reports in the literature outlining the presenting symptoms, surgical treatments offered, and outcomes related to sellar/suprasellar epidermoid cysts.

**Table 1:** Descriptive summary of sellar/suprasellar epidermoid cyst cases in the literature

Author, year	Age, sex	Presenting symptoms	Surgery (open or endonasal endoscopic transsphenoidal approach)	Total resection	Complications
Vaz-Guimaraes et al., 2018 [21]	16 years, male	Headache	Endonasal endoscopic transsphenoidal approach	NA	NA
Vaz-Guimaraes et al., 2018 [21]	18 years, female	Headache and visual loss	Endonasal endoscopic transsphenoidal approach	NA	NA
Our case	12 years, male	Visual impairment; left eye	Endonasal endoscopic transsphenoidal approach	Yes	Further left-side visual worsening (light perception) and central diabetes insipidus

## Conclusions

Sellar/suprasellar epidermoid cyst is a rare entity, especially in the pediatric age group. Since the lesion is benign and the long-term prognosis is usually good, the primary treatment goal is to achieve safe total resection in the first surgery itself as, in cases of recurrence, adhesions may make it challenging, with a high risk of morbidities. Surgical options include open and endoscopic approaches, and each option has its advantages and limitations. In our case, GTR was achieved through EEA. Due to the condition's rarity, especially in such a location and the pediatric age group, further studies comparing the various surgical options and involving long-term follow-ups are needed to help devise optimal management options in these cases.
